# A double-blinded randomized clinical trial: Topical Sahasthara extract *versus* diclofenac for treating primary knee osteoarthritis using microemulsions as a topical delivery system

**DOI:** 10.1016/j.jtcme.2026.01.002

**Published:** 2026-02-14

**Authors:** Ninnart Intharit, Arunporn Itharat, Raimar Löbenberg, Boonchana Pongcharoen, Kalyarut Phumlek, Ubonwan Saesiw, Theeraphong Ninlaor, Suchada Naknarin, Patcharaporn Muanrit, Reawfang Sriyom

**Affiliations:** a Department of Applied Thai Traditional Medicine and Center of Excellence in Applied Thai Traditional Medicine Research (CEATMR), Faculty of Medicine, Thammasat University (Rangsit campus), Klong Luang, Pathum Thani, 12120, Thailand; b Faculty of Pharmacy and Pharmaceutical Sciences, Katz Group-Rexall Centre for Pharmacy & Health Research, University of Alberta, Edmonton, Alberta, T6G 2T9, Canada; c RS Therapeutics Inc., Edmonton, Alberta, T6R 2V4, Canada; d Department of Orthopedic Surgery, Faculty of Medicine, Thammasat University (Rangsit campus), Khlong Luang, Pathum Thani, 12120, Thailand

**Keywords:** RCT, Microemulsion, Sahasthara, Diclofenac, Osteoarthritis

## Abstract

**Background and aim:**

Sahasthara (SHT), a traditional Thai herbal remedy used for anti-inflammatory and musculoskeletal disorders, lacks clinical evaluation as a topical formulation employing microemulsion (ME) technology. This study aimed to assess the efficacy and safety of a Sahasthara microemulsion (SHT-ME) compared with a diclofenac microemulsion (DF-ME) in patients with primary knee osteoarthritis (OA).

**Experimental procedure:**

Eighty-four patients aged 40–70 years were randomized to receive either 1% SHT-ME or 2% DF-ME, applied three times daily for 28 days. Efficacy was evaluated using the pain visual analogue scale (VAS), 100-m walking time, Western Ontario and McMaster Universities Osteoarthritis Index (WOMAC) scores, and global assessments. Safety assessments included physical examinations, laboratory tests, and adverse-event monitoring.

**Results:**

Both formulations significantly reduced VAS pain scores, improved walking time, and decreased WOMAC scores at days 14 and 28 compared with baseline (P < 0.05). No significant efficacy differences were observed between groups (P > 0.05). Liver and renal function, blood pressure, and biochemical parameters remained within normal ranges, and no severe or skin-related adverse events occurred in either group.

**Conclusion:**

SHT-ME provided efficacy comparable to DF-ME in relieving pain and improving mobility in knee OA. Both treatments were well tolerated and safe. These findings suggest that SHT-ME, a natural herbal alternative, and DF-ME, a synthetic drug formulation, are equally effective topical microemulsions for knee osteoarthritis management.

## Highlights

• This first clinical study compares topical SHT-ME, a Thai herbal anti-inflammatory, with DF-ME in knee OA patients.
• 1% SHT-ME achieved localized delivery with efficacy comparable to 2% DF-ME for treating knee osteoarthritis.
• Both treatments showed no serious adverse effects clinically or in laboratory safety assessments.
• SHT-ME was reformulated from oral use to topical to avoid systemic side effects like stomach discomfort or dry mouth.
• Topical SHT-ME was more effective than oral dosing, using 60 mg/day—fivefold less than oral administration.

## Graphical abstract

**Figure:**
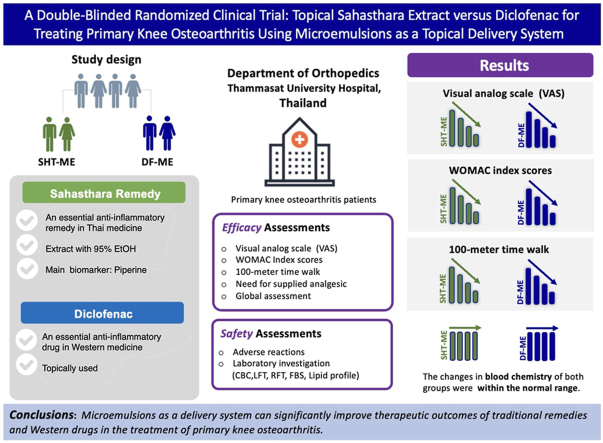

**Table:** Abbreviations:

ACR, American College of Rheumatology;	NF-kB, nuclear factor kappa B;
AEs, adverse events;	NLEM, Thai National List of Essential Medicine;
CNS, central nervous system;	NO, nitric oxide;
COX, cyclooxygenase enzyme;	NSAIDs, non-steroidal anti-inflammatory drugs;
DF-ME, diclofenac microemulsion;	OA, osteoarthritis;
HaCat, human epidermal keratinocyte line;	PGE2, prostaglandin E2;
HPLC, high-performance liquid chromatography;	RBC, red blood cells; SHT,
iNOS, inducible nitric oxide synthase;	Sahasthara; SHT-ME, Sahasthara microemulsion;
IL, interleukin;	TNF- α, tumor necrosis factor alpha;
LPS, lipopolysaccharide;	TRPV1, transient receptor potential vanilloid type 1;
KL, Kellgren-Lawrence classification;	TTM, Thai Traditional Medicine Herbarium;
ME, microemulsion;	VAS, visual analogue scale;
MMPs, matrix metalloproteinases;	WOMAC, Western Ontario and McMaster Universities Osteoarthritis Index.

## 1 Introduction

Osteoarthritis (OA) is a long-term chronic degenerative joint disease and remains a major public health concern worldwide.^1^ It is a major cause of pain and disability and severely affects the quality of life in especially elderly people.^2^ The globally prevalent cases of OA increased by 113.25% to 527.81 million from 1990 to 2019, which now affects about 7% of the global population.^3^ Patients with OA have typical clinical symptoms such as pain, joint stiffness, muscle weakness, bone enlargement, and swelling.^4^ Inflammatory cytokines, chemokines, MMPs, and other inflammatory mediators such as NO, iNOS, PGE2, IL-1, and TNF- α are released by synoviocytes, chondrocytes, other surrounding tissues, and infiltrating immune cells in the joints.^5^

Pharmaceutical interventions for OA treatment mainly aim pain relief and anti-inflammatory targets. Non-steroidal anti-inflammatory drugs (NSAIDs) have long been used as an important drug class for mild to moderate knee OA.^6^ Most of their effects are mainly due to the ability to inhibit the cyclooxygenase enzyme (COX), which synthesizes prostaglandins as a cause of pain. NSAID toxicity can be excessive with adverse events after systemic administration including gastrointestinal disturbances, CNS problems, an effect on hematopoiesis, hepatic, and renal functions.^7^ Based on the results of clinical trials with localized OA, topical NSAIDs may be equally effective as oral NSAIDs with an increased level of safety due to less systemic drug exposure.

Therefore, the development of topical formulation to treat OA is desirable to optimize efficacy and safety of such treatments. Traditional plant-based remedies provide an option for the treatment of a variety of chronic diseases.^8^ In Thailand, a traditional medication option for knee OA is known as “Sahasthara” (SHT).

SHT is a pungent-tasting remedy from “The Thai National List of Essential Medicine” (NLEM) A.D. 2006, composed of 21 herbs. This remedy has long been used as a muscle pain killer and anti-inflammatory treatment of the musculoskeletal system.^9^ An *in vitro* study reported that a SHT ethanolic extract (SHTe) had similar efficacy compared to NSAIDs. SHTe extract demonstrated potent anti-inflammatory properties by reducing COX-2 protein levels and NO production in LPS-stimulated murine macrophages (RAW246.7).^10^ No mutagenicity,^11^ or acute/subchronic toxicity was observed in animal models.^12^^,^^13^ The remedy shows a shelf life of two years at room temperature without loss of its anti-inflammatory activity.^14^ Piperine, the main biomarker delivered by piper species, has anti-inflammatory effects in human OA chondrocytes by blocking iNOS, IL-1, IL-6, TNF-, and PGE2, as well as inhibiting LPS-mediated NF-κB activation and IκB-α degradation.^15^ Recent clinical studies suggest that SHT powdered capsules could attenuate symptoms of muscle pain^16^ and osteoarthritis,^17^ and a pilot study found that SHTe cream is safe and has the potential to reduce OA knee pain within two weeks.^18^

Microemulsions (ME) are using self-emulsifying nanosized micelles to solubilize poorly soluble molecules. This topical drug delivery system has become popular in recent years. Studies showed that ME delivery systems are superior over conventional ointments or gels.^19^ However, there is no scientific research published regarding the efficacy and safety of SHT formulated as a ME.

While the development of SHT-ME was required to overcome the known limitations of oral SHT, the purpose of this study was the clinical comparison of SHT-ME to a NSAID topical formulation (DF-ME) in terms of efficacy and safety.

## 2 Method

### 2.1 Ethical approvals

The study was designed as a two-arm, double-blinded, randomized, trial to compare the safety and effectiveness of SHT-ME and DF-ME in treating primary knee OA. The protocol was approved by the Human Research Ethics Committee of Thammasat University (Medicine), Thammasat University, Thailand (Approval No. MTU-EC-TM-4-117/64), which was accepted by the Thai FDA and was filed with ClinicalTrials.gov (NCT06184685). All procedures were conducted in accordance with the ethical principles of the Declaration of Helsinki and the International Conference on Harmonisation–Good Clinical Practice (ICH-GCP) guidelines.

### 2.2 Sample size

The required sample size was calculated based on the results of a pilot study using SHTe cream in the treatment of primary knee OA^18^ as shown in the equation below. The lower level of average VAS pain scores from the previous clinical trial was 36.46 (S.D. 10.90) for SHTe cream and 29.77 (S.D. 8.86) for diclofenac. A sample size of 70 participants (35 patients/group) was estimated for an overall type I error (2-sided test) with p < 0.05, and a power of 80.^20^^,^^21^ Finally, a 20% drop-out rate of the enrolled patients was assumed, resulting in 84 patients being required to participate in the study.^21^

$$\begin{array}{l} n_{1}=\frac{{\left(z_{1-\frac{\alpha }{2}}+z_{1-\beta }\right)}^{2}\left[\sigma_{1}^{2}+\frac{\sigma_{2}^{2}}{r}\right]}{\Delta^{2}}\\ r=\frac{n_{2}}{n_{1}},\Delta =\mu_{1}-\mu_{2} \end{array}$$

### 2.3 Study design

Outpatient participants, aged between 40 and 70 years old, were recruited. They were screened for symptoms of primary knee OA at the Department of Orthopedics, Thammasat University Hospital, Pathum Thani, Thailand, between October - December 2021. A diagnosis of knee OA in this clinical trial was made using a combination of clinical and radiographic findings according to the American College of Rheumatology (ACR). Inclusion criteria for participants were those who had a pain score higher than 30 mm out of 100 mm on the visual analog scale (VAS), experiencing moderate to severe pain intensity from OA in one or both knees (patients affected by knee OA bilaterally were evaluated, and the more severely affected knee was included in the study) and had OA grade 1 to 3 (moderate/medium degree of pain) according to the Kellgren-Lawrence classification grading scale for OA severity.^22^ The subjects would be excluded if they: 1) had a history of hypersensitivity to ingredients in SHT ME or DF-ME, 2) were pregnant or breast feeding, 3) had previous surgery knee replacement or intra-articular steroid injections within 3 months of inclusion in the study, 4) were having any coexisting musculoskeletal diseases (including rheumatoid arthritis, septic arthritis, metabolic arthritis, gout, pseudogout, and traumatic arthritis) or dermatologic disorder which affected the surrounding skin of the knees, or 5) was assessed by body mass index (BMI) more than 32 kg/m^2^. The participants who were accepted to enroll in the study signed an informed consent.

### 2.4 Preparation of clinical trial materials

The composition of SHT is listed in the Thai National List of Essential Herbal Medicines^9^ and is provided in the Supplementary Materials. All the plant samples were identified by a herbalist and voucher specimens were deposited to ensure authenticity in the Thai Traditional Medicine Herbarium (TTM), Bangkok, Thailand. All plants were cleaned, suitably crushed into a coarse powder, weighed and combined according to the NLEM. SHT passed all standard quality controls of the Thai Herbal Pharmacopeia; loss on drying (moisture content), total ash content, heavy metal content, and stability under accelerated test conditions were performed. The extract was obtained by maceration with 95% ethanol for 3 days. The marc was re-extracted using the same process two more times. Then the extract was filtered, concentrated using a rotary evaporator at 40 °C under vacuum (Rotavapor R-205, Buchi, Switzerland), yielding 10.3% w/w. The ME was composed of propylene glycol monocaprylate, caprylcaproyl macrogolglycerides, diethylene glycol monoethyl ether, deionized water and 1% (w/w) of SHTe to form a ME. Finally, SHT-ME was filled and stored in 50 ml bottles before use.

High-performance liquid chromatography (HPLC) was performed to ensure the piperine content, which is the main biomarker in SHT. The acceptance criteria was “not less than” 0.09 mg/ml in the SHT-ME. A stability study showed that the SHT-ME was stable against centrifugation and freeze-thaw cycles. In addition, no changes were observed during storage for 6 months under accelerated conditions (40 °C 75% RH), piperine still had 100% remaining, which can be extrapolated to 2 years of stability at room temperature.^23^

DF-ME, which consists of 2% diclofenac sodium, was compounded according to the published instructions by RS Therapeutics Inc. (Canada)^24^ and filled and stored in the same containers as SHT-ME.

### 2.5 Clinical trial

**Fig. 1:**
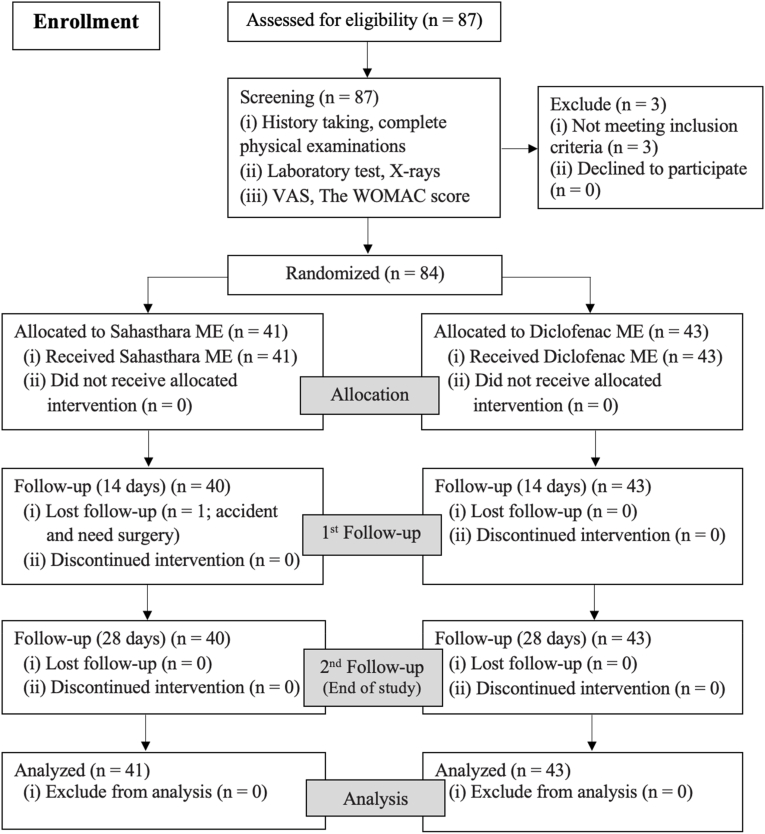
Protocol for assessments and treatment (Modified intention-to-treat). One patient in the SHT-ME group dropped out before endpoint assessment due to surgery unrelated to treatment.

The CONSORT statement was generated and submitted as supplementary material. This statement is a cornerstone of clinical trial reporting, providing a transparent and standardized means to document participant flow, ensure rigor, and support the reproducibility and trustworthiness of trial outcomes. Fig. 1 shows the clinical trial design and decision/reporting points. In brief: Following enrollment, eligible patients were given a washout period of one week with no administration of analgesics before being randomly assigned to receive one of the treatments daily for 4 weeks. A randomized code number from a non-stratified randomization list generated by computer was used. The physicians, researchers, and statisticians were blinded from the allocation of patients involved in the trial. During the study, they were instructed not to use other analgesics, including injection drugs or other medications. Following the baseline visit, patients returned to the study site after 2 and 4 weeks for follow-up assessments of safety, efficacy and study protocol compliance.

In the trial, demographic data, clinical signs and symptoms, physical examination, visual analogue scale (VAS) for pain, 100-m walk times, the WOMAC index scores (pain, physical function, stiffness), and laboratory tests (CBC, fasting blood sugar, lipid profile, liver function tests (AST, ALT, ALP), and renal function tests (BUN, creatinine clearance)) were collected as baseline data and as study data on day 14 and on day 28.

### 2.6 Drug administration

All patients were instructed to treat the knee with 2 ml, 3 times/day, for 28 days. Each of the patients received the same appearance of drug packaging, which was not revealed until data analysis was completed, or medical emergency conditions would require an unblinding. The daily dose of SHT-ME in this study was adapted from a clinical trial phase I: the irritation study of SHTe on the skin of healthy volunteers^25^ and the study of Suakitiikul et al. (2020), which used 1% SHTe cream (2 g, 3 times/day).^18^ Moreover, diclofenac sodium in the DF-ME did not exceed 160 mg per day for lower extremities as recommended.^26^ All patients were trained to use the medication directly on their knee and the surrounding tissues, spreading it evenly around until the joint was fully covered. In addition, the patients were advised not to massage the mentioned zone, instructed to wait ≥5 min before dressing, forgo showering/bathing for ≥1 h after application, avoid contact of the foam with their eyes and mouth, and wash their hands after use. All participants were allowed to use acetaminophen tablet (500 mg) as a rescue drug during the trial period. Doses of 1 or 2 tablets were permitted to a maximum of 6 tablets (3 g) per day, with ≥4 h between doses. The use of the medication was recorded.

### 2.7 Outcome measures

#### 2.7.1 Safety assessments

Safety was assessed by the incidence of adverse events (AEs), clinical examinations, and abnormalities in laboratory tests. The toxicity of the drug was evaluated. Patients were excluded from the trial following industry guidance criteria for the toxicity grading scale of the USFDA, such as creatinine more than 1.7 mg/dL, BUN more than 26 mg/dl AST, and ALT more than 2.5 times the upper limit of normal (ULN) or ALP more than 2.0 times ULN (U.S. department of health and human services, ^28^; WHO, ^27^).

#### 2.7.2 Efficacy assessments

The treatment period was completed after 28 days with the clinical and laboratory investigation follow-up assessments on the 14th and 28th day. Efficacy analyses were conducted on the intention-to-treat (ITT) population, including all randomized participants (n = 84). For participants with missing endpoint data, the last observation carried forward (LOCF) method was applied. The primary efficacy outcome measure was the WOMAC index. It is a self-administered questionnaire from the latest version of WOMAC 3.1 (the Western Ontario and McMaster Universities Osteoarthritis Index).^29^^,^^30^ The patients were evaluated by the WOMAC index in terms of three domains: pain score ranged from 0 (no pain) to 4 (extreme pain); physical function score ranged from 0 (no difficulty) to 4 (the most severe difficulty); stiffness score ranged from 0 (no stiffness) to 4 (the worst stiffness). The pain intensity was measured using the visual analogue scale (VAS), a 100-mm line representing the continuum of pain degree, arranged into 3 levels of pain: mild = 0-30; moderate = 31-60; and maximum pain = 61-100 ^31^. In addition, time to walk a straight horizontal indoor distance of 100 m (100-m time walk) and the need of the participants for taking analgesics were evaluated. Moreover, the global assessment on a 0-4 Likert scale (0: none, 4: excellent)^32^ was executed by the patients themselves at the last follow-up.

### 2.8 Statistical analysis

All statistical calculations were carried out using the IBM SPSS Statistics 26.0 software program (IBM Corporation, NK, USA). Demographic and clinical data were presented as mean values with standard deviations (SD). All efficacy analyses were conducted in the intention-to-treat (ITT) population, and missing endpoint data were handled using the last observation carried forward (LOCF) method. The differences in mean values within each group were analyzed by a paired T-test. The comparison of the radiographic grading at entry into the study was evaluated by the Chi-square test. The comparison between the two groups was analyzed by an independent samples *t*-test or a Mann–Whitney *U* test as appropriate. In addition, the primary longitudinal treatment effects were evaluated using repeated-measures ANOVA models including time (baseline, Day 14, and Day 28) as a within-subject factor and treatment group as a between-subject factor, with the time × treatment (group) interaction term used to assess differential treatment response over time. This approach accounts for the correlated structure of the repeated observations in this short-term study with minimal missing data. The comparison of the global assessment report was evaluated by the Chi-square or Fisher's exact test. Statistical significance was considered when the P value was less than 0.05 at an alpha of 0.05.

## 3 Result

A total of 87 patients were screened: 3 patients were excluded from the study; 2 patients due to KL scale grade 4 and 1 was found post-traumatic on X-ray film. The remaining 84 patients were randomized into 2 groups. A total of 43 participants were randomized to the DF-ME group and a total of 41 participants were randomized to the SHT-ME group. One participant in the SHT-ME group dropped out due to an unrelated injury requiring surgical intervention, resulting in 40 evaluable patients in that arm. Drug intervention was carried out for 28 consecutive days, starting from the first day of visiting. Accordingly, 83 (98.81%) of the patients completed the study (Fig. 1). There were no statistically significant differences between the two groups in terms of baseline characteristics (Table 1) or radiographic disease grading (Table 2).

**Table 1:** Baseline characteristics of patients in the SHT-ME and the DF-ME group.

Data	SHT-ME (n = 41)	DF-ME (n = 43)	*P* value∗
Female, number (%)	39 (95.12)	41 (95.35)	1.000^f^
Age; yrs, mean (SD)	56.98 (7.46)	56.09 (5.12)	0.531^t^
Weight; kg., mean (SD)	62.12 (10.38)	65.17 (11.04)	0.196^t^
Height; cm., mean (SD)	156.76 (6.19)	158.53 (6.52)	0.204^t^
BMI; Kg/m^2^, mean (SD)	25.25 (3.72)	25.91 (3.92)	0.428^t^
Visual analogue scale (VAS); mm., mean (SD)	56.07 (21.20)	52.60 (24.16)	0.487^t^
100-m walking time; sec., mean (SD)	79.85 (12.48)	82.25 (11.68)	0.365^t^
WOMAC index score, mean (SD)			
Pain index	9.02 (3.84)	9.05 (3.55)	0.978^t^
Stiff index	3.36 (2.06)	3.53 (1.86)	0.693^t^
Physical function index	31.80 (12.92)	31.88 (13.50)	0.978^t^
Total score	44.20 (17.82)	44.47 (18.09)	0.945^t^
Laboratory data, mean (SD)			
Glucose	99.80 (20.75)	106.37 (28.22)	0.230^t^
Blood pressure			
Systolic (mm.Hg.)	133.32 (19.95)	128.26 (18.67)	0.222^t^
Diastolic (mm.Hg.)	80.00 (11.77)	74.95 (12.48)	0.084^t^
Cholesterol			
HDL	61.00 (12.69)	62.28 (13.06)	0.650^t^
LDL	131.02 (27.94)	129.51 (44.75)	0.852^t^
Triglyceride	116.49 (54.72)	103.93 (52.88)	0.201^m^
Total Cholesterol	216.17 (32.60)	214.56 (52.85)	0.866^t^
Renal function tests			
BUN (mg/dL)	13.33 (3.92)	14.23 (4.47)	0.330^t^
Creatinine (mg/dL)	0.77 (0.12)	0.74 (0.15)	0.226^t^
Liver function tests			
AST (U/L)	29.51 (8.50)	28.63 (12.94)	0.714^t^
ALT (U/L)	35.27 (15.39)	36.42 (20.07)	0.865^m^
ALP (U/L)	87.58 (19.97)	96.00 (23.39)	0.081^t^

∗Data represent mean (SD), ∗Statistical analysis: ^f^Fisher's Exact test, ^t^Independent two-sample student's *t*-test, ^m^Mann-Withney *U* test.

**Table 2:** The radiographic grading at entry into the study.

Kellgren and Lawrence L X-ray grade	SHT-ME (n = 41)	DF-ME (n = 43)	*P* value∗
Grade 1	9	8	0.475^c^
Grade 2	20	19	
Grade 3	12	16	

∗Statistical analysis: ^c^Chi-square test.

### 3.1 Safety evaluation

The safety data of the SHT-ME group and DF-ME group at baseline, days 14 and 28 is shown in Table 3, Table 4. The blood pressure measurements, both systolic and diastolic, were not significantly different from baseline nor between the two groups. All the tests, including BUN and Cr for renal function tests, AST, ALT, and ALP for liver function tests, were performed to determine whether the given treatment might have any side events. The results showed no out-of-range laboratory measurements for all the participants and was not statistically significant between the two groups (P > 0.05). Other blood chemistry, such as fasting blood sugar and lipid profile, was in the normal range and showed no significant difference between the two groups except for HDL. Only HDL showed a statistically significant difference between the two groups (P = 0.044) but this was still within the clinically allowed range.

**Table 3:** Laboratory data in safety tissue of the patients receiving the SHT-ME and the DF-ME on baseline (Day 0), during (Day 14), and after (Day 28) the treatment of OA knee.

Data∗	Follow-up	Treatments		*P* value∗∗
		SHT-ME	DF-ME	
Blood pressure				
Systolic blood pressure (normal ≤140 mm.Hg.)	Day 0	133.32 (19.95)	128.26 (18.67)	0.661
	Day 14	132.12 (18.13)	124.81 (17.88)	
	Day 28	135.80 (20.15)	131.30 (19.17)	
Diastolic blood pressure (normal ≤90 mm.Hg.)	Day 0	80.77 (11.77)	74.95 (12.48)	0.817
	Day 14	77.83 (10.58)	72.49 (11.24)	
	Day 28	78.93 (9.91)	74.70 (11.18)	
Glucose (mg/dL) (ref.range = 90-110)	Day 0	99.80 (20.75)	106.37 (28.22)	0.490
	Day 14	100.56 (26.92)	103.23 (17.86)	
	Day 28	100.15 (26.67)	102.67 (17.95)	
Cholesterol				
HDL **(mg/dL)** **(ref.range = >40)**	Day 0	61.00 (12.69)	62.28 (13.06)	0.044
	Day 14	61.71 (14.17)	61.05 (12.72)	
	Day 28	63.76 (15.90)	61.23 (12.91)	
LDL **(mg/dL)(ref.range = <100)**	Day 0	131.02 (27.94)	129.51 (44.75)	0.734
	Day 14	129.10 (28.07)	126.07 (40.62)	
	Day 28	130.22 (34.08)	125.51 (40.31)	
Triglyceride **(mg/dL)(ref.range = <150)**	Day 0	116.49 (54.72)	103.93 (52.88)	0.381
	Day 14	107.73 (55.51)^†^	99.37 (57.08)	
	Day 28	98.93 (50.94)^††^	96.53 (53.87)	
Total Cholesterol **(mg/dL)(ref.range = <200)**	Day 0	216.17 (32.61)	214.56 (52.85)	0.497
	Day 14	215.63 (34.38)	210.79 (48.60)	
	Day 28	214.49 (37.04)	207.63 (47.00)^†^	

*Data represent mean (SD).* For within-group analyses across time, paired t-tests were applied. For between-group comparisons over time, repeated-measures ANOVA including time and treatment (group) factors was used. † Significant difference from baseline within group (P < 0.05), †† Significant difference from baseline within group (P ≤ 0.01), ††† Significant difference from baseline within group (P ≤ 0.001).

**Table 4:** Laboratory data in safety tissue of the patients receiving the SHT-ME and the DF-ME on baseline (Day 0), during (Day 14), and after (Day 28) the treatment of OA knee (continued).

Data∗	Follow-up	Treatments		*P* value∗∗
		SHT-ME	DF-ME	
Renal function tests				
Blood urea nitrogen; BUN (mg/dL)(ref.range = 7.0-18.0)	Day 0	13.33 (3.92)	14.23 (4.47)	0.530
	Day 14	12.07 (3.74)^†^	13.60 (3.61)	
	Day 28	12.83 (3.51)	13.75 (5.00)	
Creatinine (mg/dL)(ref.range = 0.7-1.3)	Day 0	0.77 (0.12)	0.74 (0.15)	0.148
	Day 14	0.76 (0.13)	0.70 (0.14)^††^	
	Day 28	0.74 (0.12)^†††^	0.69 (0.14)^†††^	
Liver function tests				
AST (U/L)(ref.range = 15-37)	Day 0	29.51 (8.50)	28.63 (12.94)	0.968
	Day 14	26.76 (10.07)	25.56 (10.22)	
	Day 28	26.12 (8.35)	24.81 (12.31)	
ALT (U/L)(ref.range = 30-65)	Day 0	35.27 (15.39)	36.42 (20.07)	0.751
	Day 14	33.95 (16.84)	35.72 (19.07)	
	Day 28	32.46 (16.31)^†^	32.77 (18.68)	
ALP (U/L)(ref.range = 50-136)	Day 0	87.59 (19.98)	96.00 (23.39)	0.060
	Day 14	87.66 (19.71)	92.91 (21.43)^†^	
	Day 28	88.78 (21.95)	92.93 (22.50)	

*Data represent mean (SD).* Within-group changes were analyzed by paired t-tests. Between-group effects over time were evaluated using repeated-measures ANOVA including time and treatment (group) factors. † Significant difference from baseline within group (P < 0.05), †† Significant difference from baseline within group (P ≤ 0.01), ††† Significant difference from baseline within group (P ≤ 0.001).

### 3.2 Efficacy of SHT-ME for treating primary knee OA

The results are summarized in Table 5 for the SHT-ME and DF-ME.

**Table 5:** The efficacy outcome of the patients receiving the SHT-ME and the DF-ME on baseline (Day 0), during (Day 14), and after (Day 28) the treatment of OA knee.

Data∗	Follow-up	Treatments		*P* value∗∗
		SHT-ME	DF-ME	
Visual analogue scale (VAS); mm	Day 0	56.07 (21.20)	52.60 (24.16)	0.861
	Day 14	35.80 (21.84)^†††^	32.21 (18.38)^†††^	
	Day 28	25.80 (20.16)^†††^	24.26 (18.11)^†††^	
100-m walking time (second)	Day 0	79.85 (12.48)	82.25 (11.68)	0.879
	Day 14	77.08 (10.55)	78.89 (8.51)^†^	
	Day 28	74.90 (10.18)^†††^	77.07 (8.85)^†††^	
WOMAC index score, mean (SD)				
Pain index	Day 0	9.02 (3.84)	9.05 (3.55)	0.602
	Day 14	6.85 (3.72)^†††^	6.81 (3.39)^†††^	
	Day 28	5.88 (4.06)^†††^	5.28 (3.63)^†††^	
Stiff index	Day 0	3.37 (2.06)	3.53 (1.86)	0.076
	Day 14	2.61 (2.11)^††^	2.09 (1.62)^†††^	
	Day 28	2.26 (2.05)^†††^	1.93 (1.44)^†††^	
Physical function index	Day 0	31.80 (12.92)	31.88 (13.50)	0.849
	Day 14	24.12 (13.93)^†††^	23.30 (11.60)^†††^	
	Day 28	22.12 (13.24)^†††^	21.16 (12.81)^†††^	
Total score	Day 0	44.19 (17.82)	44.46 (18.09)	0.702
	Day 14	33.59 (19.21)^†††^	32.21 (15.64)^†††^	
	Day 28	30.27 (18.88)^†††^	28.37 (16.75)^†††^	

*Data represent mean (SD).* Between-group longitudinal comparisons were based on repeated-measures ANOVA including time and treatment (group) factors, while within-group changes from baseline were assessed by paired t-tests. † Significant difference from baseline within group (P < 0.05), †† Significant difference from baseline within group (P ≤ 0.01), ††† Significant difference from baseline within group (P ≤ 0.001).

Both formulations significantly reduced VAS pain scores, improved WOMAC index scores across all three domains (pain, stiffness, physical function), and shortened 100-m walking times at days 14 and 28 compared with baseline (P < 0.001). The reduction in VAS pain corresponded to a shift from moderate to mild pain, aligning with commonly accepted minimal clinically important differences (MCIDs) for knee OA. No statistically significant differences between SHT-ME and DF-ME were observed for any efficacy measure (P > 0.05), indicating comparable therapeutic benefit. The pain experienced by the two groups of subjects was also found to be reduced from moderate to mild. Both groups reduced the 100-m walk times, which SHT-ME significantly reduced the time to walk on day 28, while DF-ME significantly reduced the mean times since day 14. The 100-m walking test was administered using standardized procedures; however, baseline functional capacity (e.g., cardiovascular status, gait disorders) was not systematically evaluated. These factors may have influenced performance and represent a limitation of the functional outcome assessment. Future trials should incorporate a broader set of baseline physical health metrics to contextualize mobility outcomes. In addition, among patients who did need to take acetaminophen as a rescue therapy, the patients in the SHT-ME group used an overall average of 0.45 ± 1.04 tablets while the DF-ME group used 1.09 ± 2.54 tablets, though with no statistical significance (P value = 0.334) between the groups.

The global assessment showed the overall effectiveness of both OA treatments at the end of the study (day 28). There was no statistically significant difference between the two groups. The majority of the patients in both groups have indicated scores of “very much better” and “excellent” (Table 6).

**Table 6:** Overall assessment of treatment evaluated at day 28.

Global assessment (point)	SHT-ME (n = 41) Number (%)	DF-ME (n = 43) Number (%)	*P* value^a^
0: none	2 (4.88%)	0	0.635^f^
1: mild better	0	1 (2.33%)	
2: moderate better	8 (19.51%)	10 (23.26%)	
3: very much better	18 (43.90%)	16 (37.21%)	
4: excellent	13 (31.71%)	16 (37.21%)	

a Statistical analysis: Fisher's Exact test.

### 3.3 Adverse events

The adverse events found in both groups were different: a dry mouth and throat (4.88%) in SHT-ME group and flatulence (2.33%) and dizziness (2.33%) in DF-ME group. However, there was no significant difference in the overall occurrence of adverse events between the two groups (Table 7).

**Table 7:** Adverse events of SHT-ME and DF-ME.

Adverse events	SHT-ME (n = 41) Number (%)	DF-ME (n = 43) Number (%)	*P* value^a^
**Dry mouth and throat**	2 (4.88%)	0	0.235^f^
**Flatulence**	0	1 (2.33%)	1.000^f^
**Dizziness**	0	1 (2.33%)	1.000^f^

a Statistical analysis: Fisher's Exact test.

## 4 Discussion

Sahasthara (SHT) is an alternative medicine for muscle pain and inflammation. The administration of SHT is recommended to be taken before meals, and the precautions should be used carefully in patients with hypertension, peptic ulcer, and gastric esophageal reflux disease. The adverse events of this remedy are abdominal discomfort, nauseousness, dry throat, and itchy rash.^9^ This might be caused by piperine which activates the transient receptor potential vanilloid type 1 (TRPV1), leading to a painful and burning sensation.^33^^,^^34^ The use of SHT is usually in the form of crude mixture or ethanolic extract.

The SHT-ME in this study was prepared from an ethanolic extract**,** which differs phytochemically from traditional water-based decoctions. Ethanol extraction enriches lipophilic constituents such as piperine, gallic acid, ellagic acid, plumbagin, and β-asarone, known for anti-inflammatory and analgesic effects through modulation of NF-κB, COX-2, TNF-α, and IL-6 pathways. Their synergistic actions may explain the clinical improvement observed. This extraction difference should be considered when comparing our results with aqueous SHT formulations.

A previous report found that SHTe extract has anti-inflammatory activities through the inhibition of NO and PGE-2 with IC_50_ values of 2.81 μg/ml and 16.97 μg/ml, respectively.^10^ SHTe extract at a concentration of 10 and 30 μg/mL can significantly suppress ROS production and downregulate the genes involved in NF-кB signaling in IL-1β-treated NHDFs compared with IL-1β-induced cells.^35^ Moreover, the analgesic or antiarthritic effects of piperine (100 mg/kg), the main active compound, effectively improved the symptoms of arthritic diseases with an effect comparable to prednisolone in a rat arthritis model.^36^ Although piperine was selected as a marker in the present study, there are other bioactive compounds in SHT remedy that are related to anti-inflammatory and other pharmacological activities, such as ellagic acid, gallic acid, plumbagin, and β-asarone.^37^ Ellagic acid (50 μM) could ameliorate the progression of mouse OA chondrocytes by suppressing the activation of the NF-kB pathway.^38^ Gallic acid, a metabolite of propyl gallate from *Terminalia chebula* Retz., inhibited PGE2 production at a low concentration of 25 μM.^39^ Plumbagin occurs in *Plumbago indica* L. at 15 μM reduces pro-inflammatory cytokine expression, including TNF-α, IL-6 and IL-8 in primary rat chondrocytes cells.^40^ The aqueous rhizome extract of *A. calamus*, containing β-asarone at a concentration of 10 mg/mL, exhibited negligible activity in stabilizing red blood cell membranes and inhibiting hemolysis.^41^ However, its aqueous leaf extract significantly inhibited the expression of pro-inflammatory cytokines IL-6 and IL-8 in HaCaT cells, with EC_50_ values of 106 and 99.7 ng/ml, respectively, and suppressed NF-κB activation following PGN induction.^42^ In addition, other herbs have also been associated with the treatment of OA. *I. obscura* extract, for example, can downregulate Matrix metallopeptidase (MMP) and the expressions of COX-2 and NO synthase, as well as significantly reduce elevated levels of pro-inflammatory cytokines such as IL-1, IL-6, TNF- α, and NO.^43^ While this study focused on clinical endpoints rather than mechanistic evaluation, future research should investigate the phytochemical composition of the ethanol extract and explore the potential synergistic effects of these components through both *in vitro* and *in vivo* studies.

Topical drug delivery of therapeutic agents via the skin to a specific site offers several benefits in comparison to the oral route.^44^^,^^45^ Microemulsions as topical dosage form are known for enhancing the permeation of both hydrophilic and lipophilic drugs, resulting in an improvement of the local efficacy of a drug as compared with conventional formulations such as solutions, gels, or creams.^46^ Topical remedies of 0.5% and 1% of SHTe extract have been reported to be safe for use in clinical trials.^25^ SHT was formulated into a microemulsion with 1% (w/w) of extract.

SHT-ME has anti-inflammatory activity, as demonstrated by NO inhibition on RAW 264.7 murine macrophage cell lines and COX-2 inhibitory activity with IC_50_ values of less than 10 and 25 μg/ml, respectively. Moreover, a 72-h patch test of SHT-ME in 36 healthy volunteers found no irritation or allergic reactions (unpublished data).

Topical NSAIDs are used as an alternative to oral NSAIDs. An *in vitro* study showed that 2% DF-ME had the highest release rates in comparison to other topical bases. Therefore, a DF-ME has a great potential to enhance the transdermal delivery after topical administration.^47^^,^^48^ In a previous observational study, 2% DF-ME caused about 50% reduction in VAS score from baseline, indicating that it is effective in the treatment of mild to moderate musculoskeletal pain.^19^ In our study, the daily diclofenac dose did not exceed 120 mg for the affected knee, which was less than the recommended upper dose for lower extremities.^26^ The present study employed fixed dosages of SHT-ME and DF-ME based on prior data. Dose-dependent adverse events were not assessed, and the 28-day duration limits conclusions about long-term safety. Future work should include dose-ranging trials and extended safety monitoring to evaluate cumulative effects and optimal therapeutic windows.

Three parameters were measured in this study, including the level of knee pain by VAS (mm) after the 100-m walk, the 100-m walking time, and the WOMAC index scores (pain index, stiffness index, and physical function index). The levels of knee pain in the SHT-ME group (*n* = 41) and the DF-ME (*n* = 43) were reduced significantly (*p* < 0.001) in both follow-ups (14th and 28th days). Comparison between these two groups on the VAS score showed no significant difference (*p* > 0.05) which indicated that SHT-ME is equal to DF-ME in reducing knee pain. The 100-m walking time comparison between the SHT-ME group and the DF-ME group showed that they were not significantly different (*p* > 0.05). Although the SHT-ME group was significantly reduced after 28 days of treatment (p < 0.001), the DF-ME was significantly reduced after 14 days already. The WOMAC index scores (pain index, stiffness index, and physical function index) in both groups were reduced significantly (*p* < 0.01) in both follow-ups (14th and 28th days). And when compared between the two groups, the results showed no significant difference. These results were consistent with those in the recent studies on knee pain using 100 mg of SHTe extract capsules 3 times a day, orally before meals, which showed the same efficacy on day 14th and 28th in all parameters, including the VAS score, the 100-m walking time, and the WOMAC index scores, which were equal to that of diclofenac capsules (P > 0.05).^49^ Moreover, these results were improved compared to a study on knee pain using 1000 mg of SHT powdered capsules 3 times per day, orally before meals, which showed lower efficacy than diclofenac in terms of stiffness index (P < 0.05).^17^ SHT for treating primary knee OA in the form of a crude extract requires a dose of 3000 mg/day^17^ or an equivalent ethanolic extract in capsules of 300 mg/day.^49^^,^^50^ In the present study the topical SHT-ME used only 60 mg/day of SHT extract, which is 5 times lower than the oral dose. This result shows that a lower dose can achieve the same or even better results if efficiently delivered via a ME.

For the safety evaluation, renal and liver function tests showed no significant difference between the two groups, although some variables tend to decrease from baseline. However, there was no laboratory test that changed outside the standard values. The glucose and cholesterol of both groups were not different, except for HDL. A significant difference in HDL levels between groups at baseline was observed. Although lipid profiles, particularly HDL, have been associated with systemic inflammation and joint health, this variable was not adjusted for in the outcome analysis. Future studies should control for lipid-related parameters to better assess their potential impact on treatment response. According to the study, blood pressure is one of the most concerning adverse events of SHT and therefore might affect hypertensive patients. The present study found no hypertensive effect in all patients, and this result is in line with a previous *in vivo* study of SHT (100, 300, or 1000 mg/kg/day) in hypertensive rats, which showed no effect on blood pressure.^13^ Other clinical studies of oral administration of SHT also report no effect on blood pressure.^17^^,^^18^^,^^37^^,^^50^ It can be assumed that SHT and SHTe extract, both topical and oral administered, do not affect hypertensive patients.

Our study reported no serious adverse events. No patients dropped out due to adverse events of the medications or lack of efficacy. We found no cases of flatulence and dizziness in the SHT-ME group but a few in the DF-ME group. 4.88% reported symptoms of dry mouth and throat in the SHT-ME group, which was fewer compared with the study after oral administration: 12.50% and 33.33%, respectively.^49^^,^^50^ Although most reported adverse events from oral SHT involve gastrointestinal symptoms, topical use could potentially lead to dermatological reactions such as rash or local irritation. No skin-related adverse events were reported during the 28-day trial period. This study was limited to a 28-day treatment window without extended post-treatment follow-up. As a result, the persistence of therapeutic effects and any delayed symptom recurrence were not assessed. Future trials should incorporate longer-term follow-up to evaluate the durability of pain relief and functional improvements.

This study did not collect detailed information on participants’ lifestyle, occupational activities, or exercise habits, which may influence knee OA progression and therapeutic response. Future studies should include these variables as potential covariates or stratification factors to reduce bias related to mechanical joint stress.

Although this study was not powered as a formal non-inferiority trial, the observed improvements in pain (VAS) and function (WOMAC), both of which reached clinically meaningful thresholds consistent with published MCIDs for knee OA, suggest that SHT-ME provides therapeutic benefits equivalent to topical diclofenac. This has practical implications for patient care, particularly for those who prefer herbal medicines or are unable to tolerate systemic NSAIDs due to gastrointestinal or cardiovascular risks. Therefore, SHT-ME may serve as a clinically relevant and well-tolerated alternative in the topical management of primary knee osteoarthritis. Although this study was not designed or powered as a formal non-inferiority trial, the clinical outcomes indicate that SHT-ME and DF-ME exhibited comparable therapeutic benefits in terms of pain reduction, functional improvement, and global assessment, which improved daily life activities of the participants. The absence of significant differences suggests potential therapeutic equivalence. This has practical implications, as SHT-ME may offer a natural alternative to NSAIDs, particularly for patients seeking herbal options or those at risk of adverse drug reactions to diclofenac.

While this study was conducted at a single center to establish the feasibility, safety, and preliminary efficacy of SHT-ME in comparison to DF-ME, future investigations will include multicenter trials to confirm these findings across broader and more diverse patient populations. This will enhance the generalizability and external validity of the results.

Although repeated-measures ANOVA with LOCF is appropriate in this study with a short follow-up and very low missingness (98.8% completion), future larger trials with longer observation periods will consider linear mixed-effects models, which can more flexibly address the longitudinal data structure and missingness mechanisms.

The 28-day evaluation period limits conclusions about long-term efficacy and cost-effectiveness. Nevertheless, SHT-ME achieved pain and function outcomes comparable to topical diclofenac. The economic feasibility of the herbal formulation needs to be further assessed. This randomized controlled trial demonstrates that topical SHT-ME provides pain relief and functional improvement comparable to DF-ME, while maintaining a favorable safety profile without hepatotoxic or nephrotoxic effects. The magnitude of improvement exceeded commonly referenced MCIDs, supporting the clinical meaningfulness of both treatments for the management of knee OA.

Future work will include larger non-inferiority trials and long-term safety assessments with the goal of regulatory approval and commercial availability.

## 5 Conclusion

This study evaluated the use of SHT-ME as a topical treatment for primary knee OA in comparison with a NSAID drug, DF-ME. According to the clinical trial findings, SHT-ME provides localized drug delivery with the same efficacy as DF-ME in the treatment of OA knee. Both treatments showed no negative effects on liver and renal function.

A limitation of the present study is its relatively short duration (28 days), which may not fully capture long-term safety or cumulative effects, especially in the context of chronic conditions such as knee OA. Although no severe adverse events were observed during the study period, extended clinical trials with longer follow-up will be essential to assess the sustained safety and tolerability of SHT-ME and DF-ME during prolonged use.

As a result, a natural remedy and pharmaceutical drug which were formulated as topical ME were found to be safe and effective in OA knee treatment. The SHT-ME is an alternative for patients who prefer not to take synthetic drugs. The results of this study will be used to continue the clinical development with a larger number of patients, which can lead to the approval of commercial products. This study demonstrates that microemulsions as delivery system of known traditional remedies and drugs can significantly improve therapeutic outcomes.

## Author contributions

Ninnart Intharit: methodology, investigation, formal analysis, writing - original draft. Arunporn Itharat: methodology, supervision, formal analysis, resources. Boonchana Pongcharoen: methodology, investigation, formal analysis. Raimar Löbenberg: methodology, visualization, formal analysis, resources, re-writing and editing language and grammar. Kalyarut Phumlek: methodology, investigation. Ubonwan Saesiw, Theeraphong Ninlaor, Suchada Naknarin, Patcharaporn Muanrit, Reawfang Sriyom: investigation. All authors read and approved the final version of the manuscript.

## Declaration of generative AI and AI-assisted technologies in the writing process

During the preparation of this work the author(s) used ChatGPT 4.0 in order to improve the English. After using this tool/service, the author(s) reviewed and edited the content as needed and take(s) full responsibility for the content of the publication.

## Declaration of competing interest

Raimar Löbenberg was academic supervisor of Ninnart Intharit, he is co-founder of RS Therapeutics, RS Therapeutics provided FoamaDerm microemulsion and the dispenser bottles used in the study.
